# Oxytocic Action of Placenta

**Published:** 1915-07

**Authors:** 


**Oxytocic Action of Placenta.** Curtis. Surg., Gyn. & Obs., Mch. 1915, experimented with finely divided human placenta, extracted for 24-96 hours. 16 pregnant rabbits and guinea pigs were used. Four, receiving small doses did not abort. The rest either aborted or suffered prompt delivery. Corpora lutea were found in none. Defibrinated blood from pregnant or puerperal women failed in 5 to 10 cases, results were doubtful in 2, in 3 abortion or resorption of embryo occurred. Defibrinated blood from non-pregnant women and physiologic salt solution in controls, produced no action. Extract of guinea pig placenta in physiologic salt solution was only slightly oxytocic to pregnant guinea pigs. Whole blood from healthy puerperal women administered to patients at term gave negative results in one, caused transient labor pains in one and marked labor pains in one. No toxic symptoms occurred. (Note: Very important therapeutic and equally serious ethical consequences may be looked for from this line of study).

